# Senile and Traumatic Cataract

**Published:** 1872-02

**Authors:** D. B. St. John Roosa

**Affiliations:** Clinical Professor of Diseases of the Eye and Ear in the University, and Surgeon to the Manhattan Eye and Ear Hospital, New York


ATLANTA

### MEDICAL AND SURGICAL JOURNAL.

NEW SERIES.

VOL. IXB-FEBRUARY, 1872.—NO. 11.

### ORIGINAL COMMUNICATIONS.

### SENILE AND TRAUMATIC CATARACT.

*A Clinical Lecture Delivered at the University Medical College, New York, December* 20, 1871.

BY D. B. ST. JOHN ROOSA, M.D.,

Clinical Professor of Diseases of the Eye and Ear in the University, and Surgeon to the Manhattan Eye and Ear Hospital, New York.

GENTLEMEN,—I present to you two cases, which illustrate two kinds of cataract, and which will serve as the basis of my lecture.

Cataract is any opacity of the so-called crystalline lens and its capsule, or any opacity of the capsule or lens alone. The idea, so prevalent among the laity, that a cataract is an opacity of the cornea, you will often have occasion to correct. The name *cataract* is from the old times in medicine, when knowlege was not as exact as now. It means, literally, *a running down*—the meaning being, that something which obscured vision had fallen over the lens.

Let us briefly consider the anatomy of the lens. It is this beautifully-transparent body which I now remove from the eye-ball of a sheep. It fits into the concavity of the vitreous humor, and is convex on both the anterior and posterior surfaces, but more so posteriorly. It is a true lens in shape. It is surrounded by a capsule, which is what the microscopists call a homogeneous membrane—that is to say, a membrane in which there is no definite structure. I incised this capsule in extracting the lens, when the latter was pressed out. The lens itself is ultimately composed of delicate tubes and cells, which I attempt to represent on the blackboard—tubes which are pressed together, filled with a watery fluid, and so arranged that the lens may be peeled off, layer by layer, like an onion. It is an opacity of this fluid in these tubes—or, I should say, a turbidity of this fluid—which is the proximate cause of cataract. The lens has no blood-vessels or nerves. Before birth, however, a membrane containing blood-vessels covers it and nourishes it up to that time. In very rare cases, this membrane remains after birth. Afterward, the lens must depend for its nourishment upon the adjacent ciliary body, to which it is connected, as you see in the diagram, by the so-called suspensory ligament of the lens. It obtains its vascular supply from the great vascular membrane, the choroid, by transfusion through the aqueous and vitreous humors. This suspensory ligament is an extension, as you see, from the hyaline member of the vitreous and the anterior part of the capsule of the lens. You must have considered that the lens is defenseless to resist successfully any deviation from the normal condition, which might not harm a better-nourished part of the body. This want of blood-vessels causes any impairment of the nutrition to readily affect the lens. Cataract, therefore, that is not caused by injury of the capsule or lens—that is, true cataract—is essentially an atrophy of the lens tissue. By false cataract, we mean an opacity caused by the adhesion of the iris to the lens—such as occurs in iritis not promptly treated by a mydriatic. Thus, we find that diabetes causes cataract; not that there is anything in the rapid formation of sugar in the blood that should affect the lens, but because the system is so debilitated in this disease, that the lens, poorly nourished at best, soon feels the loss of blood, and becomes atrophied and opaque.

MacKenzie, in his great treatise on the eye, enumerates some twenty kinds of cataract; but the limits of one hour do not allow a full discussion of these, and I must pass on to a discussion of the two varieties which I present to you to-day.

The first case is that of a man sixty-five years of age, from Pennsylvania, upon whom I operated four weeks ago to-morrow. He then had cataract in both eyes, and was led to the hospital, not having sight enough to go about alone, although—and this is very important in the prognosis—he could distinguish the light of a candle, through his opaque lens, in every part of his visual field. When a candle-flame cannot thus be distinguished, there must be some disease in either the vitreous, choroid, retina, or optic nerve, beside that in the lens. The removal of the lens will, then, probably, not improve the sight. In such cases, the cataract is probably secondary to changes in the vascular parts of the eye; it is an inflammatory or secondary cataract. Of course, cataract is called senile when it occurs in persons of advanced years, although comparatively young persons may have *senile* cataract; but in such cases, there are other evidences that the patient has grown old before his time—such as decayed teeth, gray hair, wrinkled skin, and so on. In all advanced age, there is some opacity of the lens, especially at the center or nucleus, where the lens tubes are packed most closely, and where all senile cataract begins; but there is not enough opacity to cause inability to read, although there may be quite enough to give a yellow appearance to the lens, on examining it by oblique light or by the ophthalmoscope. I do not operate upon patients with senile cataract, unless they are blind, or nearly so, in both eyes. The risks of failure in the result are too great to allow us to shut up a person of advanced years for the number of days sufficient to secure the success of a cataract operation, at a time when he has still sight enough in one eye to go about alone; but when such a person is beginning to go about with difficulty, then operate on the eye in which the lens is most thoroughly opaque. I can barely allude to the fact that cataracts vary much in color, and that these variations form indications as to the hardness or softness of the lens, upon which much of the success of an operation depends. If a lens be too soft, it will be very difficult to remove it wholly from the eye; and the fragments of lens matter left behind are very dangerous, and fruitful sources of inflammation.

This patient was operated upon by the method known as the one by the corneal flap, with Beer's or triangular knife, which instrument I now submit to your inspection. He was etherized; an assistant held the lids, while, in the manner that I now demonstrate, the eye-ball was fixed by forceps, and a flap made in the cornea. The capsule of the lens was then divided by a so-called cystotome, when, as the third and final step in the operation, the lens was evacuated by *gentle* pressure. No accident occurred during the operation—the iris was left intact; but a day or two afterwards, probably, a slight prolapse occurred. The eyes were then bandaged, a drop of a solution of atropine (gr. iv. aqua ℥ i) was instilled, and the patient kept in a recumbent position in bed, in a darkened room for some six days. Each day the bandage was lifted—*but the eye was not examined*—and a drop of atropine instilled between the lids. The patient has had scarcely any pain, and, after the sixth or seventh day, was very gradually allowed the use of his eye, wearing blue glasses and a shade. He now can read coarse print by the aid of a convex glass lens, to take the place of the one he has lost. His vision is expressed by the fraction 1-12, and I trust that it may be still better.

This case has had a very favorable course. But you must not expect that every one will take so pleasant a one. Ten to fifteen per cent. of total losses occur in the hands of the most experienced practitions, and many cases require a *needle* operation to remove opacities and prolifications of the capsule which were left behind. We may call it average success when eighty-five eyes out of a hundred recover useful vision—which, defined, means exactly anywhere from 1-4 to 1-20.

The old operation of depression of the lens, an operation easy to perform, is now abandoned for good and sufficient reasons—the most prominent of which is, that the lens, pushed down into the vitreous, becomes a foreign body in the eye.

There are other methods of extraction, however, than that by the corneal flap and the triangular knife of Beer. The distinguished and lamented Professor Von Grâefe, of Berlin—who probably had the largest experience in cataract of any surgeon—devised the operation which now goes under his name. The extraction is done with a narrow knife; the incision begins and ends in the sclerotica, and the iris is always excised. It is claimed that suppuration of the cornea is less likely to occur in this operation, or as a result of it. Presumptuous as it may seem, I have never been willing to admit this claim; while I do believe that the formation of capsular opacities or after-cataracts, detachment of the retina, opacities of the vitreous, are more apt to occur from this operation than from the old one, and all in consequence of the situation of the wound in scleral rather than corneal tissue. I advanced this view some three or four years ago; and only yesterday I read that Dr. Liebrich, formerly Grâefe's assistant—then of Paris, now of St. Thomas' Hospital, London—had abandoned the scleral cut for one in the cornea. Indeed, many of the advocates of the new operation have so modified it, that it is nothing more than the old operation done with a narrow knife; the name *peripheric linear* has no appropriateness in such cases.

Both of these operations require great experience, and not one of you should do them on the living subject until you have mastered all the details on the eyes of animals. Grâefe's operation is more easy of performance than the corneal flap operation, because the narrow knife is more readily manipulated, and you are allowed to make a sawing motion.

The other patient is a miner from New Jersey, who, from a premature explosion, received a charge of gunpowder in his eyes. The left eye was so injured that its removal became necessary; in the other, the lens became immediately opaque. If an attempt had been made to remove this lens by extraction, we should have found the substance very soft, and we would have been unable to remove the whole of it. The portions remaining behind would undoubtedly have caused such a severe inflammation, by their swelling and consequent pressure, that the eye would have been lost by irido-choriodeïtis, or panophthalmitis, or general inflammation of the eye-ball. I therefore undertook to remove it by the method known as

"discision," or "the method by absorption." A needle was introduced through the periphery of the cornea, the capsule was wounded, and a portion of the lens contents allowed to escape into the aqueous humor. It then became slowly absorbed; and when all the irritation in the eye-ball had subsided, the operation was repeated. Five operations of this kind have now been performed; the greater portion of the lens has been absorbed, and the patient has vision enough to go about alone, and to read the street signs. Great prudence is to be observed in the performance of this operation. If we do not wait patiently until all the irritation has subsided from one operation before we advance to another, too much lens matter may be emptied into the aqueous, excessive intraocular pressure be caused, and the eye lost from inflammation. This patient has been under my care for the last seven months. He is now to go home, and when all the loose portions of lens matter have become absorbed, it may be necessary to again use the needle.

